# Qualitative inquiry into perceptions of HIV pre-exposure prophylaxis among people who inject drugs living with hepatitis C in Seattle, WA, USA

**DOI:** 10.1186/s12954-022-00706-5

**Published:** 2022-11-01

**Authors:** Michael P. Barry, Elizabeth J. Austin, Elenore P. Bhatraju, Sara N. Glick, Joanne D. Stekler, Elyse L. Tung, Ryan N. Hansen, Emily C. Williams, Alexander J. Gojic, Eleanor I. Pickering, Judith I. Tsui

**Affiliations:** 1 Department of Epidemiology, Hans Rosling Center, School of Public Health, University of Washington, 3980 15th Ave. NE, 8th Floor, Box 351619, Seattle, WA 98195 USA; 2 HIV/STD Program, Public Health – Seattle and King County, 401 5th Ave. Suite 1300, Seattle, WA 98104 USA; 3 Department of Health Systems and Population Health, Hans Rosling Center, School of Public Health, University of Washington, 3980 15th Ave. NE, 4th Floor, Box 351621, Seattle, WA 98195 USA; 4 Division of General Internal Medicine, Department of Medicine, Harborview Medical Center, University of Washington, 325 9th Avenue, Box 359780, Seattle, WA 98104 USA; 5 Division of Allergy and Infectious Diseases, University of Washington, 1959 NE Pacific St., Box 356423, Seattle, WA 98195 USA; 6 Department of Global Health, Hans Rosling Center, School of Public Health, University of Washington, 3980 15th Ave. NE, 7th Floor, Seattle, WA 98195 USA; 7 Kelley-Ross Pharmacy Group, Seattle, WA USA; 8 Department of Pharmacy, University of Washington, H375 Health Science Building, Box 357630, Seattle, WA 98195-7630 USA; 9 Center of Innovation for Veteran-Centered and Value-Driven Care, Health Services Research and Development, VA Puget Sound, 1660 South Columbia Way, Building 101, Room 4E51, Seattle, WA 98108 USA; 10 Department of Social and Behavioral Sciences, Yale School of Public Health, 60 College Street, Suite 316, New Haven, CT 06510 USA

**Keywords:** People who inject drugs, HIV, Pre-exposure prophylaxis, Behavioral determinants, Substance use, Hepatitis C

## Abstract

**Background:**

The incidence of HIV among persons who inject drugs (PWID) in the USA has been increasing since 2014, signaling the need to identify effective ways to engage PWID in HIV prevention services, namely pre-exposure prophylaxis (PrEP). Yet, the uptake of PrEP in this population is minimal compared to other populations at risk of HIV acquisition. In this work, we sought to explore knowledge, attitudes, and perspectives of PrEP acceptability among PWID.

**Methods:**

In the context of a pilot study to explore the acceptability of pharmacy-based hepatitis C virus (HCV) treatment, we conducted semi-structured interviews (*n* = 24) and focus groups (*n* = 4, 16 participants) with people who were living with HCV and reported active injection drug use (≤ 90 days since last use). Participants were asked open-ended questions about their familiarity with and motivation to use PrEP. As part of a sub-analysis focused on PrEP, qualitative data were analyzed using a Rapid Assessment Process, where three coders used structured templates to summarize qualitative data and iteratively reviewed coded templates to identify themes. Participants also completed short quantitative questionnaires regarding drug use history and attitudes toward health concerns.

**Results:**

Forty-seven percent of participants expressed having little or no concern regarding HIV acquisition. Targeted analyses focused on HIV prevention identified three themes, which help characterize behavioral determinants of nonadoption. First, knowledge of PrEP was limited among PWID and influenced by infrequent open community discussions around HIV risk. Second, PWID perceived sexual behaviors—but not injection drug use—as a motivator for HIV risk prevention. Finally, PWID identified many individual and environmental barriers that hinder PrEP uptake.

**Conclusion:**

Among PWID, PrEP is rarely discussed and concerns about the feasibility of using daily PrEP are common. Taken with the prevalent perception that drug use is not a high risk for HIV acquisition, our findings point to opportunities for public health work to target PrEP education to PWID and to leverage other successful interventions for PWID as an opportunity to provide PrEP to this vulnerable population.

## Introduction

As the opioid epidemic persists in the USA [1], associated morbidity and mortality continue to rise. People who inject drugs (PWID) such as opioids are at increased risk of acquiring blood-borne pathogens including human immunodeficiency virus (HIV) [2, 3]. The opioid epidemic threatens progress toward goals of reducing new HIV infections to 75% by 2025 and 90% by 2030, as set by the *Ending the HIV Epidemic in the US* (EHE) plan [4]. While HIV cases have decreased among men who have sex with men (MSM) nationally, they have remained stable among PWID [5]; in Seattle, WA, USA, previous trends in declining HIV cases among PWID have stalled [6]. The 2018–2019 HIV outbreak among local heterosexual PWID [3] highlights the vulnerability of this group to rapid outbreaks of HIV seen elsewhere in the USA in recent years [2, 7, 8]. The need for targeted HIV prevention efforts in this population is clear; to meet EHE target goals and mitigate the spread of HIV associated with injection drug use, more needs to be understood about behavioral determinants that influence the adoption of PrEP and other HIV prevention measures among PWID.

Daily, oral HIV pre-exposure prophylaxis (PrEP) has become a successful cornerstone of HIV prevention efforts among MSM given its high efficacy [9, 10] and acceptability in that population [11]. Locally, in 2020, PrEP use among MSM at increased risk of HIV acquisition was quite high at 45% [6], yet uptake of PrEP among has been low PWID by comparison [12, 13]; in the 2018 National HIV Behavioral Survey, only 1% of PWID from across the USA reported they had taken PrEP [5], which mirrors local estimates [6]. Despite the clear benefits of PrEP for PWID [14], we wish to understand why uptake is low.

Previous work [12, 15, 16] has found reasons for low uptake come from system, provider, and patient levels. At the systems level, HIV PrEP medications are associated with a high price in the USA and patients and/or their PrEP provider typically must navigate complex programs to defray the cost of PrEP. These include government-sponsored programs, which vary by state, and privately sponsored programs, generally funded by drug manufacturers. On the provider level, limited qualitative and quantitative work has shown barriers including lack of knowledge and competing priorities [17, 18]. The US Centers for Disease Control and Prevention (CDC) published Clinical Practice Guidelines for PrEP in 2021 [9], which recommends PrEP patients visit their providers between two and four times annually for therapy maintenance. Evidence from prior work, however, reflects continued access issues for PWID in the context of high maintenance treatment programs such as the one recommended by CDC [19–22].

Among the various qualitative studies that explored perspectives on, beliefs about, and acceptability of PrEP among PWID [19–22], researchers have found limited PrEP knowledge, frequent misinformation around PrEP medication formats and efficacy, and a disconnect between perceived and actual HIV risk levels among PWID [19–21]. Existing behavioral theories, such as the theoretical domains framework [23, 24], point to the multidimensionality of behavior change, including aspects of knowledge, beliefs, capabilities, social identity, and environment. Importantly, the behavioral theory emphasizes the need to clearly specify determinants of behavior, so as to inform appropriate behavioral change strategies [25]. While prior work has explored the perspectives of PWID on PrEP, additional work is needed to characterize the behavioral determinants of PrEP uptake in this population [13].

To contribute to the current state of literature with a better understanding of how to effectively engage PWID living with HCV—a group particularly fit for linkage to PrEP given that HCV is generally transmitted through unsafe injecting behaviors that are also linked to HIV transmission [8, 26]—in PrEP and HIV prevention services, we examined knowledge, attitudes, and beliefs about HIV prevention and PrEP among and the potential behavioral determinants that could inform future interventions to improve PrEP uptake.

## Methods

As part of the formative evaluation stage of a larger study to assess the feasibility of a community-pharmacy model to deliver HCV treatment to PWID, we conducted semi-structured, qualitative interviews (*n* = 24 interviews) and focus groups (*n* = 4 focus groups, 16 participants) with PWID (*n* = 40) in the Seattle area. We offered the option of individual or group-based interviews to support participants’ preferences, recognizing that participants might feel comfortable discussing sensitive topics in different ways. We also utilized individual and group-based interview formats to increase the richness of data gathered by triangulating individual and group reporting of shared lived experiences [27]. We approached recruitment with a combination of purposive and snowball sampling methods [28] where potential participants were sought at agencies offering services to people living with HCV and/or PWID. We identified participants at substance use disorder (SUD) clinics (*n* = 3), medical case management (CM) agencies specific to hepatitis C (*n* = 19), and low-income housing and homeless service centers, using purposive sampling to ensure the sample reflected a diverse mix of participants from different access points for services, as well as to ensure traditionally underrepresented groups were well or overrepresented. To reach participants who may not engage with service agencies where we actively recruited, we invited participants to refer their social contacts who may qualify (i.e., snowball sampling) and displayed recruitment fliers in public spaces such as bus stops and telephone poles in areas where PWID are known to congregate. The eighteen participants recruited outside of SUD clinics and CM agencies were recruited through these efforts. Since data analysis (described later) occurred concurrently with recruitment, recruitment was informed by and determined to be adequate when saturation of participant perspectives was met and interviews no longer produced new learnings.

Participants were eligible if they were at least 18 years old, reported injecting substances in the past 90 days, reported living with untreated HCV, provided informed consent to the study, and could speak and write in English. Eligible, interested persons were given the option to enroll and, if accepted, completed an informed consent process with a member of the research team. The consent process was done in writing initially but, after the implementation of COVID-19 safety protocols, was conducted verbally. All activities were reviewed and approved by the University of Washington Institutional Review Board.

Data collection occurred between January 7 and July 17, 2020. Participants chose to participate in either an individual interview or a focus group. Interviews and focus groups lasted 30–60 min. Prior to the interview or focus group, participants were asked to complete a brief survey, which included questions about demographic information, drug use behaviors, and attitudes toward health needs. Participants responded to attitudinal items using a 5-point Likert scale.

A semi-structured interview guide was developed with input from several subject matter experts including addiction medicine providers, qualitative researchers, pharmacists, and public health practitioners. The guide asked open-ended questions about knowledge, attitudes, and beliefs about HIV PrEP and HIV prevention. Specifically, the interviewer asked participants if they had ever heard of PrEP or medications to prevent HIV. If participants had no or limited awareness of PrEP, the interviewer gave a brief description of purpose, format, and efficacy of PrEP medications. Next, the interviewer asked questions related to community awareness of PrEP, prior experiences with seeking care for HIV prevention, and beliefs about the acceptability of taking PrEP in its current, once-daily format. The interviewer also asked about other aspects of seeking Opioid Use Disorder (OUD) and HCV-related care, which are reported elsewhere [29, 30]. Interviews and focus groups were conducted in person in January and February 2020. In March 2020, COVID-19 safety policies were implemented by the University of Washington and partner agencies and in accordance, staff conducted visits remotely using HIPAA-compliant Zoom or phone for the remainder of the study. All participants received a $40 cash or gift card honorarium for participating. Participants in remote visits were mailed gift cards only.

Qualitative interviews and focus groups were audio recorded and professionally transcribed and combined for analysis. We used the Rapid Assessment Process (RAP) to analyze qualitative data for the parent study as well as for this targeted sub-analysis. RAP is an approach to qualitative inquiry that uses triangulation and iterative data analysis to develop an understanding of data, and is particularly useful for formative evaluation contexts, such as for this study, where results are needed quickly to inform program design [31–33]. During the analysis for the parent study, two independent coders (MB and EA) reviewed and summarized interview and focus group transcripts using structured templates which included coded sections for PrEP awareness, attitudes, and beliefs. Each completed RAP template included a combination of summarized learnings from the qualitative data and participant quotes to ensure “thick description” [34], which were reviewed by the qualitative study lead (EW) to ensure alignment in the coding and to resolve discrepancies between coders. During this analysis, the team identified potential emergent themes related to HIV prevention that warranted a targeted sub-analysis; thus, a third independent coder (EB) coded the transcripts again to ensure all data were accurately reflected, using the same RAP template, however, only focusing on the topic of HIV prevention and PrEP. RAP templates from all three coders were then combined and used to iteratively summarize learnings and identify themes. No notable differences were noted in themes between the interview and focus group data sources. Themes were presented and refined by the core qualitative investigative team (MB, EA, EB, EW, and JT) until consensus was met between all researchers. Data from the brief quantitative surveys were cleaned and analyzed using descriptive statistics in the *RStudio* statistical analysis package [35]. Quantitative and qualitative data were triangulated during the analysis process, particularly in relation to participant attitudes toward HIV risk.

## Results

Twenty-six (65%) participants were male, 21 (53%) were white, and seven (18%) were Hispanic/Latino. The median age was 37 years, and most participants (80%) were unstably housed or homeless. Fifteen (38%) participants report using a combination of heroin and methamphetamine at the last injection episode, 27 participants (68%) reported daily injection over the past 30 days, and 35 (88%) had injected in the week preceding their study visit. The median number of years since the first injection episode was 11.5, and the median number of injection episodes on a given day when injecting was 4. When asked about their level of concern for HIV acquisition, 17 (47%) participants cited being “not concerned” or “a little concerned” and 16 (44%) participants reported being “very” or “extremely concerned.” Table 1 presents the demographic, social history, and attitudinal characteristics of the sample.

**Table 1:** Demographic characteristics of PWID living with HCV

Characteristic	Overall (*n* = 40)
Age (Median, IQR)	37 (32–45)
Gender	
Male	26 (65%)
Female	13 (33%)
Non-binary (assigned male at birth)	1 (3%)
Race	
American Indian/Alaska native	7 (18%)
Asian	1 (3%)
African-American/Black	1 (3%)
White	21 (53%)
Multiple Races	5 (13%)
Other race(s)	5 (13%)
Hispanic ethnicity	7 (18%)
Housing status	
Stably housed	8 (20%)
Not stably housed	32 (80%)
*Drug(s) used at last injection episode*	
Single drugs	
Heroin, alone	14 (35%)
Methamphetamine, alone	4 (10%)
Combinations of drugs	
Heroin and methamphetamine	15 (38%)
Heroin, methamphetamine, and fentanyl	4 (10%)
Heroin, methamphetamine, and cocaine	1 (2%)
Heroin, methamphetamine, and methadone	1 (2%)
Heroin, methamphetamine, cocaine, and fentanyl	1 (2%)
Days injected in past month (Median, IQR)	30 (25–30)
Injecting episodes on an average day (Median, IQR)	4 (3–4)
Years injecting drugs (Median, IQR)	11.5 (5–18)
Years aware of HCV diagnosis (Median, IQR)	2 (0.5–7)
Level of concern about HIV acquisition among participants	
Extremely concerned	12 (30%)
Very concerned	4 (10%)
Moderately concerned	3 (8%)
A little concerned	8 (20%)
Not at all concerned	9 (22%)
Refused	1 (2%)
Missing	3 (8%)

Qualitative analysis identified three themes that characterize the perspectives of PWID on PrEP and potential behavioral determinants of nonadoption, which are described below and are presented with salient quotes that reflect the respective themes.

### Theme 1: knowledge of PrEP is influenced by community discussion

When asked about their awareness of PrEP, many participants had limited knowledge, evidenced by the following examples.

I think I heard it [PrEP] at a AA meeting before… that you take one shot every three months or something to help… with HIV. [X006].

I haven’t really heard anything other than reading the flyers for it [PrEP] on the walls around here and there. And only a couple places so far. But that’s all I know about it... [H024].

The lack of familiarity with PrEP among participants is reflected in the absence of greater community discussion about PrEP. Many participants described that reluctance to discuss PrEP as a community was possibly rooted in privacy concerns:

I don’t really know if anybody does use it. It’s kind of like a privacy thing, you know. And I think—I’m sure people do, but I don’t know who is… It’s kind of not my business… [X009].

… people like to crack jokes about it [HIV]. But, I mean, it’s not like an openly common conversation, where you just walk up and people are like, ‘Oh, you got HIV.’ … it just depends on the group’s maturity, or the specific person. [X015].

As a result, participants described that PrEP and HIV prevention more broadly is also “just not talked about” among PWID [X017]:

In my circle of people, they don’t even bring it [PrEP] up. They might bring up hep C treatment or hep A/B treatment, stuff like that, but I never hear about it in my main circle of friends. Just a few select ones, that’s it. Back when I first used, people were more concerned with catching HIV than hep C [X001].

Lastly, as participants reflected on the lack of knowledge within their community, they pointed to the need for community-level education that worked to address the privacy barriers*.* One participant suggested that *“It’s [PrEP is] easy to get if you know about it. If you don’t know about it, then you’re not going to get it” [X002].*

### Theme 2: motivation for PrEP use varied by risk behavior

As participants reflected on the lack of open discussion around HIV and PrEP, they shared a broader sense that community members had variable and sometimes low perceived risk for HIV acquisition.

Some people talk about it [HIV] freely and other people don’t want to talk about it at all. I don’t really hear people talking about it as much as they used to, or they don’t seem as concerned about it anymore. They should be... back in the ‘80s and ‘90s, people were more scared. People aren’t as scared about it [HIV] anymore. [X020].

Some participants expressed feeling that their risk of HIV was low either because they were heterosexual or because they do not share injection equipment with others. In general, participants referenced a common attitude that sexual activity is more strongly associated with HIV risk than injection drug use is:

I mean I don’t—I don’t need it [PrEP] personally. I’m not also sexually active right now, though, either. [X015].

I would be all for it [using PrEP], if I was putting myself [at risk] – I don’t share needles at all … And I’m not sexually active … I’m for it [PrEP], though, as long as it doesn’t hurt you. But I guess it wouldn’t be any worse than the things I put in my body right now [X012].

One participant who engaged in sex work cited sexual exposure as their primary motivator for using PrEP:

I’m not sure what to think about PrEP. But I was on it because I’m a working girl. And I want to do everything to prevent myself from getting HIV.... [E003].

Still, other participants generally believed sexual risk for HIV was specific to the MSM community, and not the community of PWID:

I think it’s more like either the people that have HIV or the people that are gay or lesbian or whatever, those are the people that are more familiar with it [PrEP] than anyone else [X002].

For instance, participants often did not mention the potential benefits PrEP might confer directly to them and instead focused on its benefits for MSM:

… I have a lot of friends who are gay males and I just know that the world is pretty scary in the way they are sexually active … like I said it’s [PrEP] overall a good thing [E009].

Another participant described that “most people don’t know about it [PrEP], but unfortunately it seems to be more of a ‘unless you’re gay, I shouldn’t have to worry about this drug,’ kind of thing” [X010]*.*

### Theme 3: perceived individual and environmental barriers hinder PrEP uptake

Participants expressed several concerns that speak to the feasibility and acceptability of PrEP among PWID. First, when asked about perspectives on taking PrEP, participants expressed concern that it would not be realistic to take a medication every day:

Being out here on the streets and taking a medication to prevent a disease that we don’t have is going to, in my opinion, cause problems, because I’m not going to go out of my way to take it every day….. [H024]

Further, participants characterized their use of preventive healthcare services like PrEP as a low priority, compared to meeting basic needs (e.g., food, a place to sleep), acquiring drugs, and accessing healthcare services that were perceived as being more critical:

I probably would want to take care of the hep C first. And not really like take anything else with it... And it’s not that I'm against taking it [PrEP] at all. I would just want to make sure that I got the hep C taken care of … But I don’t want to mix a whole bunch of medications [X009].

Lastly, one participant that had experience with PrEP cited side effects as a barrier to uptake:

I've been on PrEP before, but it’s kind of scary, because I had renal failure last year. And I had to go through dialysis. I almost had a transplant. And I don’t know if it’s because of PrEP, I don’t know, because I was on PrEP when that happened. And I stopped using it. [E003]

## Discussion

This qualitative study aimed to characterize the behavioral determinants that may influence PrEP use among persons with HCV who inject drugs. The learnings from this study highlight that limited community-level knowledge, variable perceived risk and motivation, and lack of environmental support may all contribute to poor uptake of PrEP for PWID. The determinants align with existing theory related to behavior change, mainly that behavior change is predicated on an individual’s capabilities (e.g., knowledge and skills), motivation (e.g., attitudes and social norms), and opportunities (e.g., physical and social resources) to take on a new behavior [24, 36]. Interestingly, learnings from our study demonstrate that for PWID, these behavioral determinants related to PrEP and HIV prevention are closely intertwined with community-level norms and constraints. These data also highlight some potential conflict, as when surveyed, close to half of the participants indicated strong concerns for HIV risk, yet during interview discussions, participants reflected ambivalence toward PrEP uptake, in part influenced by their perspectives on community norms. When asked about levels of concern for HCV-related complications, a similar proportion (53%) of participants cited concern [30]. These findings reinforce and expand on prior studies, emphasizing the need to leverage community-level education, particularly through peer-to-peer networks, as an effective strategy to increase knowledge, attitudes, and beliefs around PrEP use [22, 37].

We found that PWID perceive their own risk of HIV acquisition to be low, associating HIV risk not with drug use but rather with sexual risk, particularly that of MSM. This is consistent with prior studies [12, 19, 38, 39] and reinforces the need to more carefully frame HIV alongside HCV as infections associated with injection drug use. Multiple studies have demonstrated the benefits of targeting HCV treatment among people with co-occurring HIV and HCV [40–42]. For example, a survey of 540 PWID living with HIV found that having a community member talk about their experiences receiving HIV care was positively associated with an increased uptake in HCV care [37]. There are therefore uncapitalized opportunities to align efforts for HIV and HCV prevention and treatment.

PrEP, in its oral form wherein high levels of adherence are necessary to prevent HIV acquisition [43], poses a barrier for PWID, particularly for those with unstable housing or who have other barriers to maintaining regular medication storage and use. Low-barrier, walk-in sites are often acceptable alternatives to appointment-only, office-based services and have proven successful in providing primary care to PWID in Seattle [44, 45]. Opportunities for examining daily, oral PrEP adherence support lie in the success of offering HCV treatment and SUD medications to PWID in a directly observed therapy setting such as opioid treatment program and in replicating those models addressed earlier in the manuscript [46–48]. Of note PrEP medications are clinically safe to take along with OUD treatments (e.g., buprenorphine and methadone) [49]. Still, it is recognized that PWID are not a monolith and individuals in this population have varying preferences for care delivery formats [29, 50].

Further opportunities exist in long-acting injectable cabotegravir as PrEP which was approved for use in the USA by the Food and Drug Administration in late 2021. Injectable PrEP may address some of the barriers to daily, oral PrEP uptake and adherence cited by participants in this study [51]. It is not known how effective long-acting PrEP is for people whose HIV acquisition risk is primarily injection drug use; this team of authors expects that, as with oral PrEP, there will be a substantial delay in clinical studies specific to injectable PrEP for PWID. Our inferences, however, are limited as when we developed our qualitative interview guide, injectable PrEP was under study. Therefore, our guide focused only on oral PrEP.

It is important to acknowledge that qualitative work, by nature, is not intended to be broadly generalizable and our work around PrEP specifically was an exploratory and preparatory component of a larger study of an intervention to improve access to essential medication for PWID. As our work was inductive and exploratory, we asked open-ended questions about PrEP with few clarification questions beyond gaining a preliminary understanding of participants’ knowledge, attitudes, and perceptions about it. This provided us with a starting point for future work on interventions to effectively link PWID communities with PrEP. Our research builds incrementally on prior research by focusing on a subset of PWID who are living with HCV, who may represent a particularly high-risk group for acquiring HCV, and for whom there may be a “window of opportunity” to educate and offer PrEP as they engage for HCV care. Future research efforts should be channeled toward developing interventions to initiate and maintain PWID on PrEP. Recent works from large cities in the USA [23, 24] and Scotland [25] have found that PrEP delivery models for persons who use drugs may be successful short term (in one study, half of patients remained on PrEP after 6 months), though they require intensive resource allocation to be so and medication adherence remains suboptimal.

There are a number of limitations of this research in addition to those listed above. Of note, as we did not formally collect information about sexual orientation, we could not comment on whether individual participants were also MSM (or otherwise at elevated risk of sexual HIV acquisition) unless they volunteered such information in their interview or focus group. It is notable that, at the time of data collection in early 2020, the incidence of HIV among PWID locally had been trending down since the 2018 outbreak [6]; still, we suspect that the aforementioned outbreaks of HIV among PWID in recent years across the USA highlight the unpredictability of HIV incidence in this group and motivate the need for strong HIV prevention programs, which include PrEP provision, targeted at PWID. Our qualitative findings may not be transferable to other cities or regions, given Seattle’s progressive policies and law enforcement practices concerned with PWID and others living unhoused, relative to elsewhere in the USA. While our sample reflected some of the diversity among PWID in Seattle, many participants in the study had similar lived experiences—specifically, they were largely people who used drugs daily and had been using drugs for the long term. PrEP’s implications for groups such as those who intermittently inject drugs, who are new to using injection drugs, or who use drugs largely in the context of sex (such as MSM who inject drugs) may be different than those among our sample. Other limitations include the focus on English-speaking sample and potential for social-desirability bias to influence participants’ responses, particularly in the context of focus groups.

This work adds to the existing knowledge base on how to improve PrEP uptake among PWID, with a focus on persons with HCV who may be amenable to models of care that offer PrEP along with other potentially life-saving medication for HCV and overdose. Results suggest a need for public health agencies to develop HIV risk and PrEP-specific education campaigns tailored to communities of PWID in order to normalize PrEP use—an approach that has been successful among other populations [52], as well as models of care delivery to deliver PrEP to PWID.

## Data Availability

The datasets used and/or analyzed during the current study are available from the corresponding author, Dr. Judith I. Tsui, upon reasonable request.
